# Association between caffeine intake and erectile dysfunction: a meta-analysis of cohort studies

**DOI:** 10.1186/s41043-024-00645-w

**Published:** 2024-09-28

**Authors:** Mehdi Karimi, Omid Asbaghi, Kimia Kazemi, Fatemeh Maleki Sedgi, Ensiye Soleimani, Hanieh Keikhay Moghadam

**Affiliations:** 1 Bogomolets National Medical University (NMU), 13, T. Shevchenko Blvd, Kyiv, 01601 Ukraine; 2 Research Center of Cancer, Shahid Beheshti University of Medical Sciences (SBUMS), Tehran, Iran; 3 Department of Food Science and Technology, Ayatollah Amoli Branch, Islamic Azad University, Amol, Iran; 4 Department of Nutrition, Faculty of Medicine, Urmia University of Medical Science, Urmia, Iran; 5 Department of Community Nutrition, Faculty of Nutrition, Tabriz University of Medical Sciences, Tabriz, Iran; 6 Department of Nutrition Science, Varastegan Institute for Medical Sciences, Mashhad, Iran

**Keywords:** Erectile, Caffeine, Coffee, Caffeinated beverages, Cohort

## Abstract

**Background:**

Erectile dysfunction (ED) is a common condition with various contributing factors, including lifestyle and dietary habits. Caffeine, a widely consumed stimulant, has been linked to multiple physiological effects on vascular function and hormonal balance that might influence sexual function. This meta-analysis aims to evaluate the association between caffeine intake and the risk of ED by analyzing data from cohort studies.

**Methods:**

A systematic search was conducted across PubMed, Web of Science, Scopus, and Embase databases, and a manual search was conducted on Google Scholar for studies on the relationship between caffeine intake and ED in adult men. The search included observational studies published up to April 1, 2024. Four cohort studies were included, and their data were extracted and analyzed by STATA version 18.

**Results:**

Four included cohort studies comprised 51,665 cohort members. The study population included adult males, on average, aged 18 to 80. The results indicate that there was no significant relationship between coffee consumption and the risk of ED (relative risk [RR] = 0.94, 95% CI: 0.86–1.03; *p* = 0.999).

**Conclusions:**

The current evidence suggests no significant relationship between caffeine intake and ED, but limited studies limit conclusions. Future research should focus on larger sample sizes, standardized outcome assessments, and different dosages and forms of caffeine consumption.

## Introduction

Erectile dysfunction (ED) is a medical condition characterized by the persistent inability to achieve and maintain a penile erection firm enough to perform satisfactory sexual intercourse. This disorder, along with premature ejaculation, is considered the most common form of male sexual dysfunction [1, 2]. ED affects around 1–10% of adults under 40 and around 30–50% of men between 40 and 70 years old. It is estimated that there will be 322 million cases of ED worldwide by 2025 [2, 3]. ED is a complex condition influenced by various vascular, hormonal, neurological, and psychological factors. Risk factors include sedentary lifestyle, obesity, smoking, excessive alcohol consumption, drug abuse, insulin resistance, diabetes, atherosclerosis, hypertension, and endocrine disorders. These factors contribute to the development and progression of ED, emphasizing the importance of healthy behaviors and controlling underlying health conditions [2–5].

Caffeine intake has become a topic of interest among researchers due to its ubiquitous usage and physiological effects, among other lifestyle factors being studied. Caffeine is a commonly consumed psychoactive compound that has an impact on both the central nervous system and cardiovascular system [6]. Caffeine is renowned for its stimulating properties and is frequently included in coffee, tea, and other drinks and edibles [7]. Caffeine generally functions by inhibiting adenosine receptors, which promote sleep and relaxation. This leads to heightened alertness and a decrease in the experience of exhaustion. Coffee and its primary component, caffeine, have potential health advantages as it contains abundant antioxidants and anti-inflammatory compounds [8, 9].

A plausible biological basis links caffeine to vascular function, including its impact on erectile function, given the penis’s high vascularity. Research indicates that caffeine in coffee can elevate testosterone levels [10, 11]. Additionally, caffeine may improve ED by upregulating cavernous cyclic guanosine monophosphate (cGMP), which relaxes cavernous smooth muscle and enhances blood flow through penile arteries [12, 13]. This process could potentially alleviate ED. Some studies suggest that moderate caffeine consumption might offer protective benefits against ED by improving endothelial function and boosting nitric oxide production [12, 14]. Despite these facts, other research warns that caffeine could have acute adverse effects on cardiovascular health, particularly impacting endothelial function, which may counteract its potential benefits for erectile function [8, 15–18]. The impact of coffee consumption on urological diseases, particularly ED, is still uncertain [17, 19, 20].

This research seeks to find the association between caffeine intake and the risk of ED through a systematic review and meta-analysis of cohort studies. Understanding this relationship is crucial for guiding dietary recommendations and lifestyle modifications aimed at improving men’s sexual health and overall well-being.

## Method

### Methodology

The current meta-analysis followed the Preferred Reporting Items for Systematic Reviews and Meta-analyses (PRISMA) guidelines to ensure adherence to the standard methodology for meta-analyses [21]. 

### Literature search strategy

A comprehensive search was conducted in PubMed/MEDLINE, Web of Science, Scopus, and Google Scholar using a combination of Medical Subject Headings (MeSH) terms for caffeine and ED. The guidelines and nomenclature provided by the National Center for Biotechnology Information (NCBI) utilized these MeSH terms. The search encompassed literature published up to April 1, 2024, and was restricted to studies published in English. Two reviewers independently evaluated each article, with any discrepancies resolved through discussion (see Table 1).

### Eligibility criteria

The inclusion criteria for studies in this meta-analysis were as follows: (1) caffeine intake (caffeine supplementation, coffee, tea, caffeinated beverages); (2) adults older than 18 years old men; (3) cohort studies design of the study. Studies were excluded if they were designed as case-control studies or other non-cohort study designs or lacked sufficient data. Two authors conducted independent literature screening, and any discrepancies were resolved through discussion until a consensus was reached (see Table 1).

**Table 1:** Search strategy adopted in the present systematic review and meta-analysis

Search Strategy	Details
**Search string**	“caffeine” OR “coffee” OR “caffeinated” AND “erectile” OR “erection” OR “penile” OR “erectile dysfunction” OR “penile dysfunction” “erectile disorders” OR “penile disorders”
**Databases**	PubMed/MEDLINE, Web-of-Science, Embase, Scopus, manual search in Google Scholar
**Inclusion criteria**	‐ P (patients/population): general population/patients suffering from erectile dysfunction ‐ I (intervention/exposure): caffeine supplementation, coffee, tea, caffeinated drinks/beverages. ‐ C (comparisons/comparators): coffee consumers versus non-consumers; different kinds of coffee (caffeinated/decaffeinated) ‐ (outcome): incidence of erectile dysfunction ‐ S (study design): Cohort study
**Exclusion criteria**	Experimental studies investigating in vitro or animal models. Study design: editorial, commentaries, expert opinions, letters to the editor, review articles, original non-cohort studies, and articles with insufficient details.
**Time filter**	None (from inception)
**Language filter**	None (any language)

### Data extraction

Following the full-text review, the selected papers were included for data extraction. Two investigators independently extracted data from each eligible study. Data extraction was conducted using a standardized documentation form, capturing the following parameters: the last name of the first author, publication year and country, sample size, participant age, BMI (body mass index), the incidence of ED, and details regarding the amount and type of caffeine consumed. Two investigators independently extracted data from each eligible study to ensure accuracy and consistency.

### Quality assessment

To evaluate the quality of the included studies, two researchers independently appraised the appropriateness of the research questions tested and the methods employed. Any disagreements were resolved through consensus between the researchers. This process ensured a comprehensive evaluation of study quality.

### Statistical analysis

Data analysis for this study was conducted using STATA version 18. Statistical significance was determined using a threshold of p-values less than 0.05. Cochran’s Q and I-squared tests were employed to assess between-study heterogeneity, categorized as follows: I² < 25% indicating no heterogeneity, I² = 25–50% indicating moderate heterogeneity, and I² > 50% indicating high heterogeneity. Corresponding 95% confidence intervals (CIs) were also calculated to provide a range of plausible values for the true effect size [22].

### Publication bias

Publication bias was assessed using Begg’s funnel plot to examine its symmetry visually. Additionally, Egger’s regression asymmetry test and Begg’s adjusted rank correlation were used to evaluate funnel plot asymmetry formally. In cases where publication bias was detected, the trim-and-fill method was applied. This method estimates potentially missing studies due to publication bias and adjusts the overall effect estimate accordingly.

## Results

### Study characteristics

**Fig. 1:**
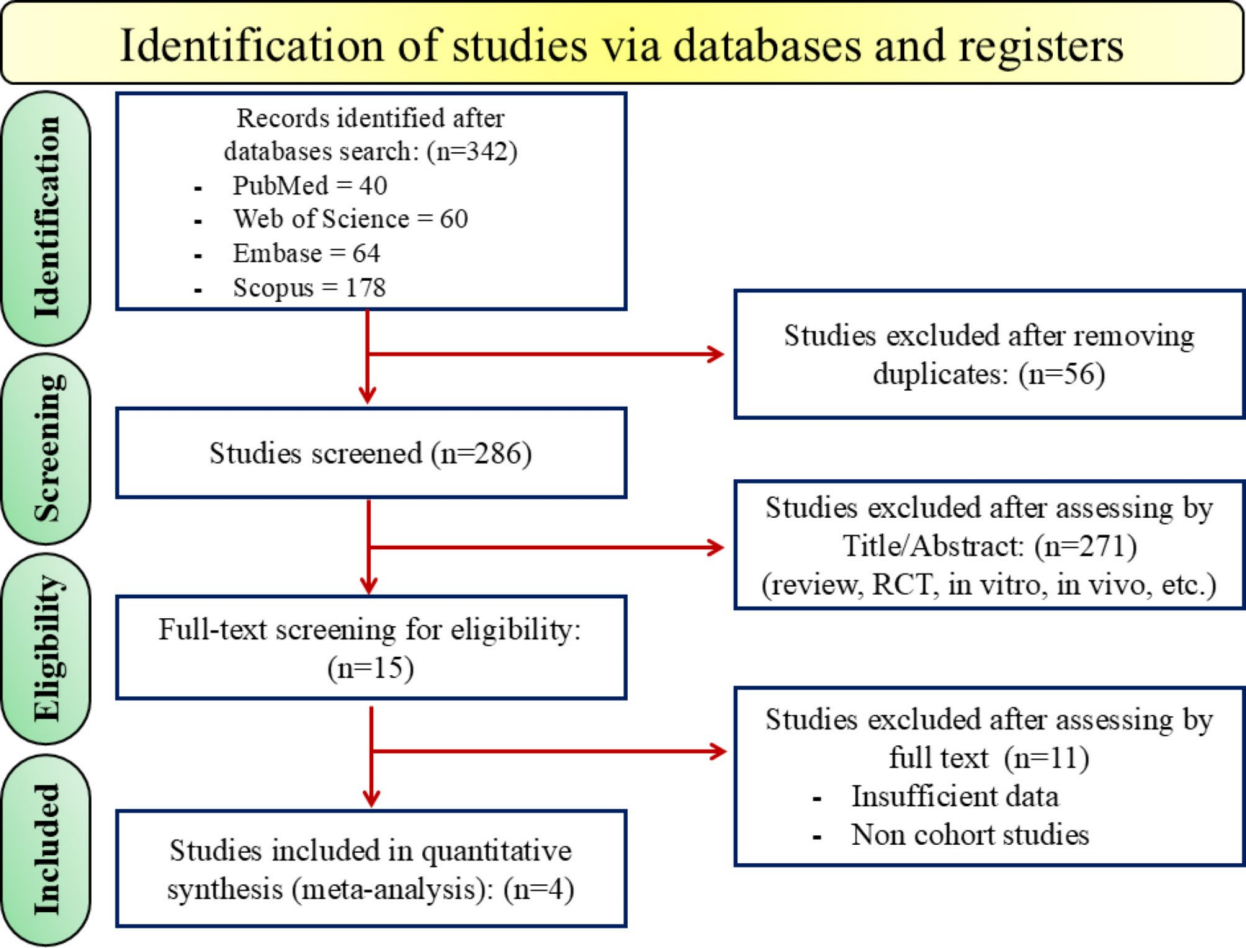
PRISMA flowchart diagram

A total of 342 studies were identified through database searches, with the following distribution: PubMed (*n* = 40), Web of Science (*n* = 60), Embase (*n* = 64), and Scopus (*n* = 178). After removing 56 duplicates, 286 studies remained for screening. Following a review of titles and abstracts, 271 studies were excluded due to reviews, randomized controlled trials (RCTs), or studies conducted in vitro, in vivo, etc., leaving 15 studies for full-text eligibility screening. After a thorough full-text assessment, 11 studies were excluded due to insufficient data or for not being cohort studies. Ultimately, 4 cohort studies were included in the quantitative synthesis (meta-analysis) (see Fig. 1).

### Included cohorts

This meta-analysis included 51,665 men from four cohort studies [8, 16–18]. The articles were published between 2004 and 2017. Three of these studies [8, 16, 18] were conducted in America, while one was conducted in Finland [17]. The duration of follow-up for incident ED cases ranged from 3 to 24 years (see Table 2).

**Table 2:** The characteristics of studies that evaluated the association between erectile dysfunction risk and increased caffeine intake among adult men

Author/year	Country	Study Design	Sample size	Follow-up period	Age (y)	Exposure	Received dose	OR, RR	Main findings
Lopez et al. 2017	USA	Cohort	21,403	1998–2010	40–75	Coffee	65% of participants = at least one cup of coffee, 11% of participants = 4 or more cups of coffee daily	1.00 (0.90, 1.11)	Long-term coffee intake was not associated with the risk of ED in a prospective cohort study.
Cassidy et al. 2016	USA	Cohort	25,096	1986–2010	40–75	Coffee and Flavonoids	2–3 cups/ day	0.91 (0.85, 0.97)	Coffee and tea, which are specific flavonoid-rich foods, might reduce ED incidence.
Lopez et al. 2015	USA	Cohort	3724	2001–2004	< 20	Caffeine and Caffeinated beverages	8-375 mg/day	0.69 (0.44, 1.07)	Caffeine intake reduced the odds of prevalent ED, especially an intake equivalent to approximately 2–3 daily cups of coffee (170–375 mg/day). This reduction was also observed among overweight/obese and hypertensive, but not among diabetic men.
Shiri et al. 2004	Finland	Cohort	1442	1994–1999	50–70	Coffee	< 5 cups/day	1.10 (0.70, 1.70)	Coffee consumption has no impact on the incidence of ED. Obesity and current smoking increased the incidence of ED. Level of education, marital status, urban/rural place of residence, amount of alcohol, and coffee consumption had no impact on the incidence of ED.

* BMI; body mass index; RR: relative ratio; OR: odd ratio

### Caffeine and ED

The results of this meta-analysis indicate that there was no significant relationship between coffee consumption and the risk of ED (relative risk [RR] = 0.94, 95% CI: 0.86–1.03). The I-squared statistic was 34.9%, indicating moderate heterogeneity.

**Fig. 2:**
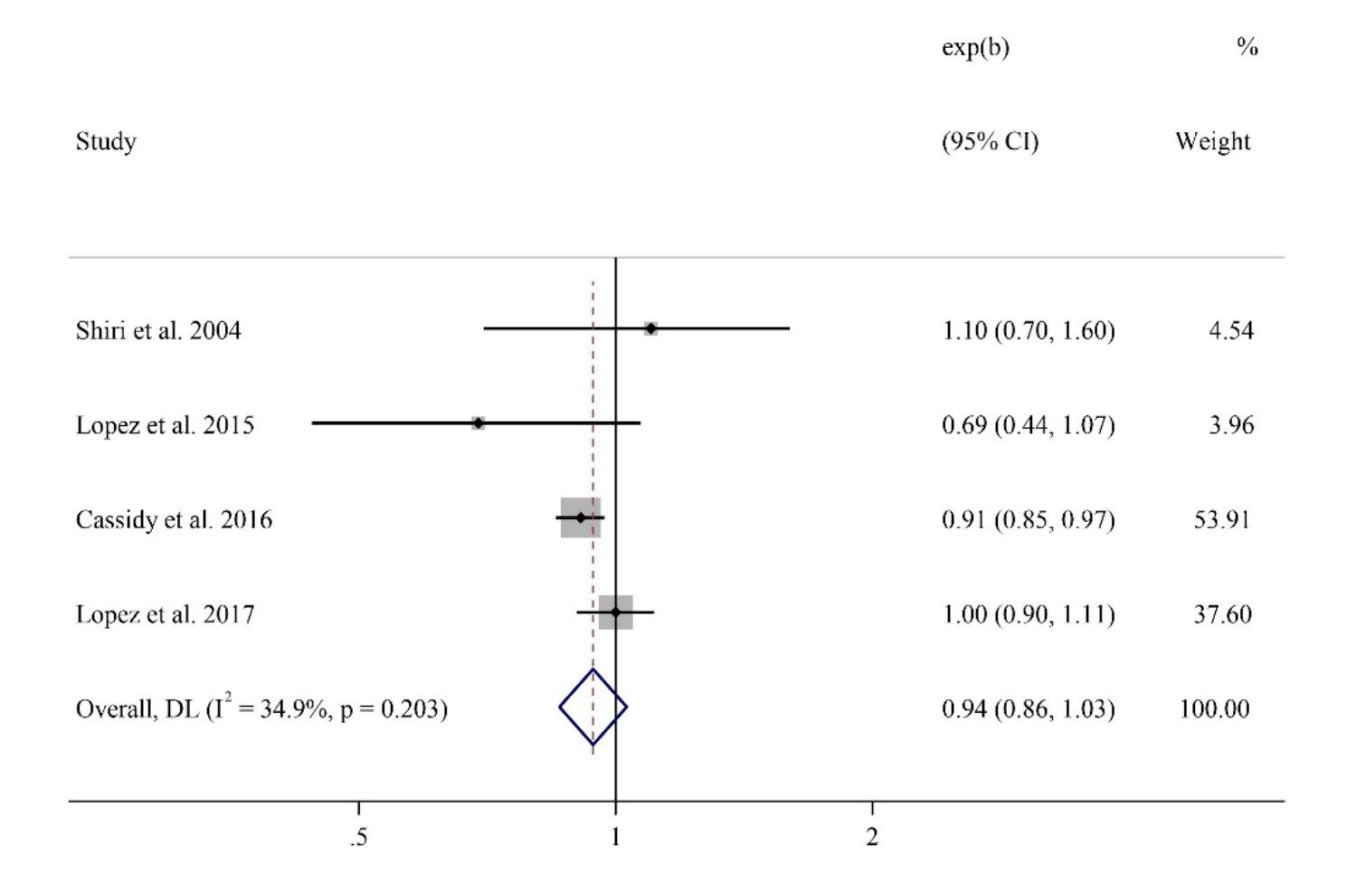
Forest plot of RR of ED for coffee consumption

Figure 2 demonstrates the results of the four included cohort studies. Each study’s RR and 95% confidence interval are shown. The RR ranged from 0.69 to 1.1, with overlapping confidence intervals. The summary diamond represents the overall pooled RR of 0.88 (95% CI: 0.86–1.03) from the meta-analysis, indicating that coffee consumption was not significantly associated with the risk of ED.

**Fig. 3:**
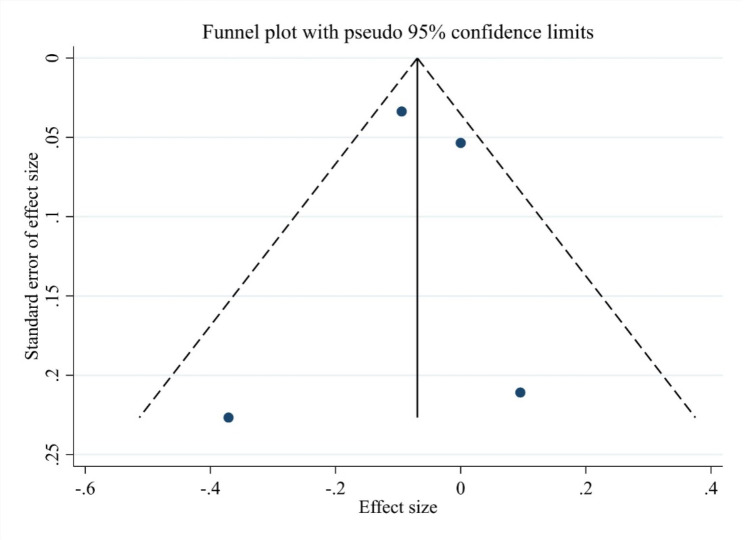
Egger’s funnel plot for the risk of ED

The distribution of studies included in the meta-analysis. The symmetrical distribution of studies suggests a low risk of publication bias. This finding is further supported by Egger’s regression test, which yielded a p-value of 0.999, indicating no evidence of minor study effects or publication bias among the included studies Fig. 3.

## Discussion

This meta-analysis of cohort studies aimed to discover the association between caffeine intake and the risk of ED in men. To the best of our knowledge, this is the first systematic review and meta-analysis uniquely focused on this aim. In this current study, we did not find a significant correlation between caffeine intake and the risk of developing ED, which adds a new dimension to the ongoing debate about the effects of caffeine on sexual health. Caffeine consumption has been linked to various potential health benefits, including improvements in cognitive function, enhanced physical performance, and a possible reduction in the risk of chronic diseases [23].

Caffeine is an alkaloid in familiar dietary sources like coffee and tea. Coffee is abundant in caffeine, antioxidants, and anti-inflammatory compounds, making it a valuable source of these beneficial components [5, 24–27]. It has been suggested that it offers beneficial effects in preventing or managing chronic diseases. Previous studies highlighted the role of ED in health. Experience of unsatisfactory sexual performance can lead to stress, affect self-confidence, and contribute to relationship problems, ultimately affecting men’s overall quality of life [28, 29].

A few studies have indirectly mentioned the association between caffeine intake and ED, with mixed results. Allen et al. [30], in their meta-analysis study, studied the factors affecting sexual dysfunction and health-related lifestyle. Regarding caffeine intake, they reported that there is no significant association between caffeine intake and ED. In another study, Lopez et al. [8] found that higher caffeine intake (equivalent to 2–3 daily cups of coffee, or 170–375 mg/day) was associated with reduced odds of ED in men. This may suggest a beneficial effect of moderate caffeine consumption on the vascular system and erectile function. Conversely, Shirai et al. [31] observed improvements in erection frequency, firmness, confidence, and overall satisfaction in men with ED who consumed 40 mg/day of caffeine. This supports the idea that caffeine supplementation might enhance certain aspects of erectile function, at least in men already experiencing ED. A prospective cohort study by Lopez [16] in 2018, however, found no association between long-term coffee intake and the risk of ED. This aligns with our findings and suggests that the duration and pattern of caffeine consumption may not significantly influence the risk of ED.

Additionally, Shiri et al.‘s population-based follow-up study indicated no significant impact of coffee consumption on the occurrence of ED [17]. In a large observational study involving 21,403 participants, Lopez et al. also found no significant correlation between overall or regular coffee consumption and ED [16]. These studies further corroborate our results and highlight the consistency across diverse population-based research.

Interestingly, our findings contrast with those of Shaeer et al. [32], who reported a significant correlation between caffeine consumption and increased prevalence of ED in a large cross-sectional study. Similarly, another study by Lopez et al. [8] found that caffeine intake was associated with reduced odds of prevalent ED, particularly with 2–3 cups of coffee per day, which contradicts our findings.

Discrepancies in the impact of caffeine on ED across different studies can be attributed to several factors, including variability in study design, such as sample size, population characteristics, and assessment methods. Differences in how caffeine intake is measured, whether through self-report or objective measures and the type and amount of caffeine consumed can also contribute to inconsistent findings. Additionally, variations in ED assessment tools, population characteristics, and confounding variables like lifestyle factors and overall health play a role. A key factor might be differences in habitual caffeine consumption across different nations and cultures, which can influence the observed effects on ED. The quality of studies, including potential biases and methodological limitations, further impacts the results [33]. Lastly, individual biological variability in caffeine metabolism may lead to different effects on ED among individuals [34].

Besides, animal studies have indicated that caffeine consumption may affect erectile function [13, 35]. In an animal study on rats conducted by Yang et al. [13] Caffeine consumption improved the erectile function of diabetic rats by up-regulating cavernous cGMP. An animal study showed that caffeine consumption may have an erectogenic effect on cavernous tissue and enhance erectile function in rats by increasing cavernous cyclic guanosine monophosphate (cGMP) activity [35].

One suggested mechanism for this phenomenon is the increased availability of nitric oxide, which improves endothelial function [36]. Additionally, caffeine can stimulate prostacyclin production in cavernosal tissue [37]. It is worth noting that human corpus cavernosum tissue can produce prostacyclin (PGI2), a vasodilator and potent platelet aggregation inhibitor. Given that penile erection in humans relies on parasympathetic stimulation, the vasodilatory action of PGI2 may contribute to the initiation and maintenance of an erection [38]. It acts as a nonselective phosphodiesterase inhibitor; by inhibiting phosphodiesterase, caffeine increases the levels of intracellular cyclic guanosine monophosphate (cGMP) and cyclic adenosine monophosphate (cAMP) [39].

One limitation of our study is the relatively small number of cohort studies available for inclusion, which may impact the generalizability of the findings. We also acknowledge that including only cohort studies rather than placebo-controlled randomized trials could introduce inherent bias into the analysis. Additionally, we recognized that the included studies hold significant weight in the overall analysis. The substantial heterogeneity in study design and total caffeine consumption within these cohorts may significantly impact the data and overall conclusions. Despite these limitations, a key strength of our study is that it is the first meta-analysis to specifically focus on the association between caffeine intake and ED, providing a comprehensive synthesis of the available evidence. Our rigorous methodology and adherence to established meta-analytic procedures further enhance the reliability of the results.

## Conclusion

The current evidence suggests no significant relationship between caffeine intake and ED. However, it is essential to note that the available studies may be limited in number, which hinders the ability to draw robust and definitive conclusions. Further research involving larger sample sizes and more comprehensive study designs is needed to provide more robust and reliable findings. Specifically, future studies should focus on standardized outcome assessments and consider various dosages and forms of caffeine consumption to establish a clearer causal relationship between caffeine intake and ED.

## Data Availability

No datasets were generated or analysed during the current study.

## Abbreviations

ED: Erectile Dysfunction
IIEF-EF: International Index of Erectile Function
EAU: European Association of Urology
cGMP: Cyclic guanosine Monophosphate
cAMP: Cyclic adenosine Monophosphate
CIs: Confidence Intervals
RR: Relative Risk
